# Key barriers and solutions for decarbonizing Egypt’s construction sector

**DOI:** 10.1038/s41598-026-37170-1

**Published:** 2026-03-30

**Authors:** Sara Harb, Ibrahim S. Abotaleb, A. Samer Ezeldin

**Affiliations:** Department of Construction Engineering, School of Sciences and Engineering, The American University in Cairo, P.O. Box 74, New Cairo, 11835 Egypt

**Keywords:** Engineering, Materials science, Structural materials

## Abstract

Decarbonization in the construction industry demands a cyclical, collaborative approach involving architects, engineers, material suppliers, contractors, and clients. Each stakeholder plays a pivotal role in fostering continuous innovation and improvement to reduce the industry’s carbon footprint. Despite increasing attention on low-carbon materials and sustainable practices, the construction sector remains a significant source of emissions. Existing research often overlooks the unique challenges faced by developing countries like Egypt, failing to address key barriers or align them with actionable and context-specific solutions. The goal of this research is to identify and analyze the financial, technological, and regulatory challenges obstructing decarbonization efforts in the construction sector. To address this, the research bridges this gap through a comprehensive, multistep methodology. Initially, an industry survey was conducted with the following objectives: (1) identifying and prioritizing 32 barriers to decarbonization; (2) providing a clear prioritization of these challenges by evaluating their significance and impact on the decarbonization process; (3) collecting and analyzing rankings to understand who stakeholders believe should take the lead on carbon reduction; and (4) exploring stakeholders’ priorities and perspectives, focusing on understanding their views on barriers and potential solutions. Second, different statistical analysis tests and methods (i.e., internal, and external reliability, statistically significant differences, evaluation metrics, clustering analysis, and heatmap analysis) were employed to analyze the results and draw conclusions. Based on a total of 125 responses and 32 decarbonization barriers, the findings reflected that the top three barriers adversely affecting decarbonization construction projects included: (1) perception of higher construction costs; (2) limited market which prioritizes cost and speed over reducing embodied carbon; and (3) high initial investment costs for low-carbon technologies. Barriers were categorized into three clusters based on criticality, with 56% requiring immediate action. This study not only ranks barriers but also aligns them with actionable solutions, offering insights to guide decision-makers, researchers, and practitioners. It provides a roadmap for overcoming decarbonization challenges and advancing sustainable construction practices in Egypt.

## Introduction

Given the complexity of built environment projects, decarbonization requires a cyclical and collaborative approach involving various stakeholders, architects, engineers, material suppliers, contractors, and clients. According to Arogundade, Dulaimi, & Ajayi^1^, each stakeholder contributes to a continuous loop of improvement and innovation aimed at reducing the overall carbon footprint of construction. While Victoria & Perera^2^ identified the key role played by architects in selecting low-carbon materials to replace high carbon-intensive ones, thereby reducing the total embodied carbon of a project. This decision feeds into the cycle as it impacts material suppliers, who respond by providing sustainable, low-carbon materials and innovating new solutions to further minimize carbon emissions. These suppliers also enhance the cycle by offering transparency regarding the carbon content of their products, enabling architects and clients to make more informed, sustainable choices. According to Walker & Walker^3^, clients are pivotal in driving decarbonization by adopting Early Contractor Involvement (ECI) models. This approach strategically brings contractors into the project early to leverage their expertise, maximizing decarbonization potential from the outset. Engineers then ensure that the chosen low-carbon materials and construction techniques are applied efficiently, maintaining the building’s structural integrity and performance while minimizing its environmental impact. This cyclical process of selecting, supplying, implementing, and optimizing sustainable practices and materials creates a robust feedback loop where each stakeholder’s actions reinforce the others, continuously driving down the carbon footprint of built environment projects.

There are many barriers to decarbonization in the construction sector; including but not limited to, economic, technical, awareness, legislative, resources and collaboration. Many of these barriers have been addressed by researchers and construction professionals. Giesekam et al.^4^ highlighted main barriers as perceptions of high cost, ineffective allocation of responsibility, industry culture, and the poor availability of product and building-level carbon data and benchmarks. Similarly, Arogundade, Dulaimi, & Ajayi^1^ classified the barriers into 15 critical categories, with their factor analysis revealing four main factors: resources and prioritization, policy and standards, risk and commitment, and awareness and complexity.

In the local context, data analysis by Mohamed et al.^5^ identified six key barriers to decarbonization in the building sector in Egypt: the lack of mandatory regulations, the high cost of certain measures, client demands, inadequate market awareness and knowledge, insufficient resources, and the complexity of various decarbonization procedures. It is important to note that these barriers primarily pertain to the formal construction sector. A significant portion of construction activity in Egypt, as in many developing economies, occurs within the informal sector. This sector likely faces distinct or amplified versions of these barriers (e.g., even more severe resource constraints, minimal regulatory oversight, limited access to information/training) while potentially operating largely outside frameworks designed to address decarbonization. Understanding the specific challenges and dynamics of decarbonization within Egypt’s informal construction sector remains a critical gap in the current literature.

The literature also highlights opportunities for achieving decarbonization, such as involving professionals earlier in the supply^1,4^, effectively utilizing whole-life costing, and adjusting contract and tender documents^4^. Other studies review decarbonization strategies for the building materials industry, focusing on steel and cement, to reduce CO_2_ emissions^6,7^. According to Sbahieh et al.^7^, these reviews also assess alternative materials that offer a lower environmental impact compared to traditional options. Jaglan and Korde^8^ argue that without government-enforced climate legislation, the decarbonization process is unlikely to accelerate effectively.

## Knowledge gap and research objectives

Despite the growing literature on decarbonization, low-carbon materials, and sustainable practices in the construction industry, it remains a major source of carbon emissions. Much of the current research lacks context-specific insights for developing countries like Egypt, often overlooks specific barriers, and fails to align these barriers with actionable solutions. To better understand the challenges, several studies have surveyed industry experts. Mohamed et al.^5^ designed a questionnaire based on 25 decarbonization measures from the Green Pyramids Rating System (GPRS), EDGE, and the Regional Roadmap for Buildings and Construction in Africa, identifying eight key measures for the Egyptian sector. Giesekam et al.^4^ classified barriers to adopting low-carbon materials into institutional, economic, technical, and knowledge-based categories, underscoring the need for stronger regulatory frameworks to support widespread adoption. Arogundade, Dulaimi, & Ajayi^1^ investigated carbon reduction barriers in UK construction using a survey based on a comprehensive literature review and pilot studies, analyzed through mean ranking and factor analysis. The NEPC report emphasizes decarbonization via product outcomes, design, specification, construction, and procurement changes^9^. Shell International BV^10^ highlights embodied carbon challenges, drawing insights from nearly 100 experts across the construction sector.

Table 1 synthesizes and compares this study’s contributions with prior research, serving two primary functions. First, it demonstrates the comprehensiveness of this work. While existing studies often address isolated components, such as identifying barriers or proposing general solutions^1,4^ this research is unique in its integrated treatment of all five key elements: barrier identification, alignment with actionable solutions, distribution of stakeholder responsibility, exploration of stakeholder priorities, and pinpointing of critical issues for targeted action.

Second, the table underscores how this study addresses a significant contextual gap. Much prior research overlooks the distinct challenges of developing economies. In the Egyptian context, earlier work has focused almost exclusively on the formal construction sector, neglecting the substantial informal sector where challenges like severe resource constraints and limited regulatory oversight are likely amplified. By directly engaging with local industry stakeholders to capture their perspectives, this research provides tailored, practical strategies that bridge the gap between identified barriers and feasible decarbonization pathways for carbon-intensive markets.

**Table 1:** Literature contribution of this research.

Reference	Identify barriers	Align barriers with actionable solutions	Distribute responsibility among stakeholders	Explore stakeholder priorities and perspectives	Identify critical issues for targeted efforts
NEPC (2021)	–	–	X	–	X
Shell International BV (2023)	X	X	–	X	X
Arogundade et al., (2024)	X	–	–	–	–
Giesekam et al., (2015)	X	–	X	X	X
Mohamed et al., (2024)	X	–	–	–	X
Shell International BV (2023)	X	X	X	X	X
OneClick LCA (2024)	X	X	–	–	–
World GBC 2019)	X	X	X	–	–
This research	X	X	X	X	X

The goal of this research is to identify and analyze the financial, technological, and regulatory challenges obstructing decarbonization efforts in the construction sector. The associated objectives are to: (1) Identify and analyze the financial, technological, and regulatory challenges obstructing decarbonization efforts; (2) Provide a clear prioritization of these challenges by evaluating their significance and impact on the decarbonization process; (3) Collect and analyze rankings to understand who stakeholders believe should take the lead on carbon reduction; and (4) Explore stakeholders’ priorities and perspectives, focusing on understanding their views on barriers and potential solutions. While there has been growing awareness of embodied carbon in construction, it remains a niche topic. The widespread estimation and mitigation of embodied carbon are likely to take years to become standard across all sectors and organizations. Consequently, this study did not aim to recruit a representative sample of Egypt’s construction industry. Instead, it focused on individuals with significant experience in low-carbon materials, including stakeholders from five key groups: design and development, sustainability and compliance, engineering and execution, and manufacturing and supply chain. The perspectives of these early adopters are crucial, as their motivations and experiences will shape the future adoption of low-carbon practices across the industry. Their insights are particularly valuable in crafting regulatory strategies and guidance to drive the broader adoption of sustainable construction materials.

## Literature review

The construction industry faces numerous barriers to adopting sustainable practices and alternative materials, particularly in developing economies like Egypt. This section explores these barriers in two key areas: (1) the adoption of alternative materials, and (2) innovation within the industry. It examines challenges such as the lack of databases for green materials, limited awareness, outdated regulations, and insufficient financial incentives. By analyzing findings from various studies, the review highlights the complexities of transitioning toward low-carbon practices and offers insights into the systemic changes needed to overcome these obstacles.

### Barriers to the adoption of alternative materials in the construction industry

Mohamed et al.^5^ note that there is currently no database for all available green building materials in the local market; as such, users lack access to comprehensive information needed to compare and score the real performance, health, environmental, and economic impacts of different materials. Zhang and Canning^11^ argue that this issue is further complicated by the insufficient marketing and dissemination of information about new materials to practicing engineers. Additionally, the absence of full-scale demonstration projects makes it difficult to gauge the true potential of these materials. The authors suggest that these challenges could be addressed through the addition of design guidance, combined with more effective marketing strategies and enhanced stakeholder engagement. Additionally, a study by Salama and Hana^12^ investigating sustainable construction awareness among professionals in the United Arab Emirates (UAE) revealed that stakeholders identified a lack of awareness about the benefits of carbon reduction as the primary obstacle to adopting green building practices. This finding is corroborated by research in a similar regional context; a study by AlSanad^13^ of the Kuwaiti construction industry also identified a lack of awareness as the primary barrier to implementing sustainable construction (SC), highlighting a common challenge across the Gulf region.

Giesekam et al.^4^ conducted a survey primarily involving architects, engineers, and sustainability consultants, yielding 32 complete responses. The findings revealed that many respondents find it challenging to assign responsibility for material selection and embodied carbon reduction to a single stakeholder, given the numerous actors involved in project decisions. The consensus among most interviewees was that the focus should be on creating a continuous chain of responsibility to ensure that sustainable solutions are effectively implemented, and the collective responsibility should be integrated into the contract structures for all stakeholders. In addition, another barrier is the difficulty of securing insurance for new and reused materials. These materials often lack comprehensive performance data, established testing standards, and proven long-term durability, making insurers hesitant to provide coverage. This uncertainty can increase premiums or even make insurance unattainable, adding financial risk for developers and contractors. Consequently, the adoption of alternative materials is often discouraged, hindering the broader implementation of sustainable practices, despite their potential environmental advantages.

### Barriers to innovation in the construction industry

Arora et al.^14^ observe that the construction industry is marked by fragmentation, risk aversion, and a heavy dependence on suppliers, noting that many firms operate with small teams and have limited research and development (R&D) capabilities. Osmani and O’Reilly^15^ and Pinkse and Dommisse^16^ highlight that construction companies struggle to comprehensively evaluate new materials and technologies due to their reliance on specific human capital and their focus on temporary, unique projects with varying stakeholders. This leads to a project-by-project learning approach, where knowledge is built from individual experiences rather than systematic learning, resulting in a reluctance to adopt unfamiliar technologies and materials. Additionally, Moncaster at al^17^ consider that the transfer of knowledge is impeded by ineffective transfer of information from academic research to industry practice. The averseness to innovate is worsened by outdated regulatory standards do not keep pace with technological advancements, leading firms to continue using traditional materials^14,18^. Williams and Dair^19^ argue that effective and up-to-date legislation is crucial, as the absence of regulatory requirements results in overlooking environmental issues. Furthermore, legislations are widely recognized as a key motivator for the construction industry to tackle environmental challenges, with many companies driven by the potential competitive edge gained through proactive adaptation to forthcoming regulations^15^.

In the Egyptian context, the regulatory landscape for sustainable construction is anchored by several key instruments. The Egyptian Code for Improving the Efficiency of Energy Use in Buildings^20^ is the primary mandatory standard, focusing on reducing operational energy consumption through the building envelope, lighting, and HVAC systems. Furthermore, Law No. 4 of 1994 (Environmental Law) mandates Environmental Impact Assessments (EIAs) for major projects, addressing broader environmental concerns like pollution and waste management. Water scarcity has also driven specific Water Conservation Regulations promoting efficient fixtures and reuse. While these frameworks represent critical progress, they exhibit significant gaps that align with the barriers identified in this study. Crucially, ECP 306–2017 focuses solely on operational energy and does not address embodied carbon, directly correlating with barrier B16 (Lack of clear carbon reduction regulation by the government). Similarly, the enforcement mechanisms (B17) for these codes can be inconsistent, and there is no single regulating agency for carbon accounting (B19), leading to a fragmented approach. This regulatory landscape effectively addresses energy use and specific environmental impacts but creates a void regarding the comprehensive carbon footprint of construction materials and processes, a central concern of this research.

Despite recent market shifts, the prevailing financial climate has limited demand for innovative low-carbon buildings^18,21^ as clients typically prioritize project cost, timelines, functionality, and aesthetics over environmental considerations^22^. This creates a clear dilemma, as while the construction industry must reduce embodied carbon emissions from materials, widespread adoption of alternative materials remains challenging in a sector traditionally resistant to innovation. This challenge is compounded by the absence of financial incentives for clients to drive this transition. Kumaraswamy, Welege, & Pan^23^ highlight that incentives are crucial not only for material and equipment manufacturers but also for owners and investors to adopt low-carbon buildings. Common constraints to low-carbon building adaptation include insufficient investment capital, lack of incentives for the building material and equipment market, and inadequate support from government and financial institutions for owners and developers. These factors collectively hinder the progress towards widespread adoption of low-carbon building solutions in various regions. Additionally, the ineffective translation of useful research outcomes into technology innovations, policy initiatives, and industry practices is a significant and prevalent constraint. This gap further impedes the advancement of low-carbon building solutions, despite the availability of valuable research.

To provide a conceptual lens for analyzing the interconnected nature of these barriers, this study applies a Multi-Level Governance (MLG) framework. MLG is particularly apt for decarbonization, which requires coordinated action across supranational climate goals, national policy, local enforcement, and non-state actors (e.g., private firms, NGOs). This framework helps structure the analysis of how barriers at one level (e.g., lack of national policy) create challenges at others (e.g., inconsistent local enforcement).

## Research methodology

**Fig. 1:**
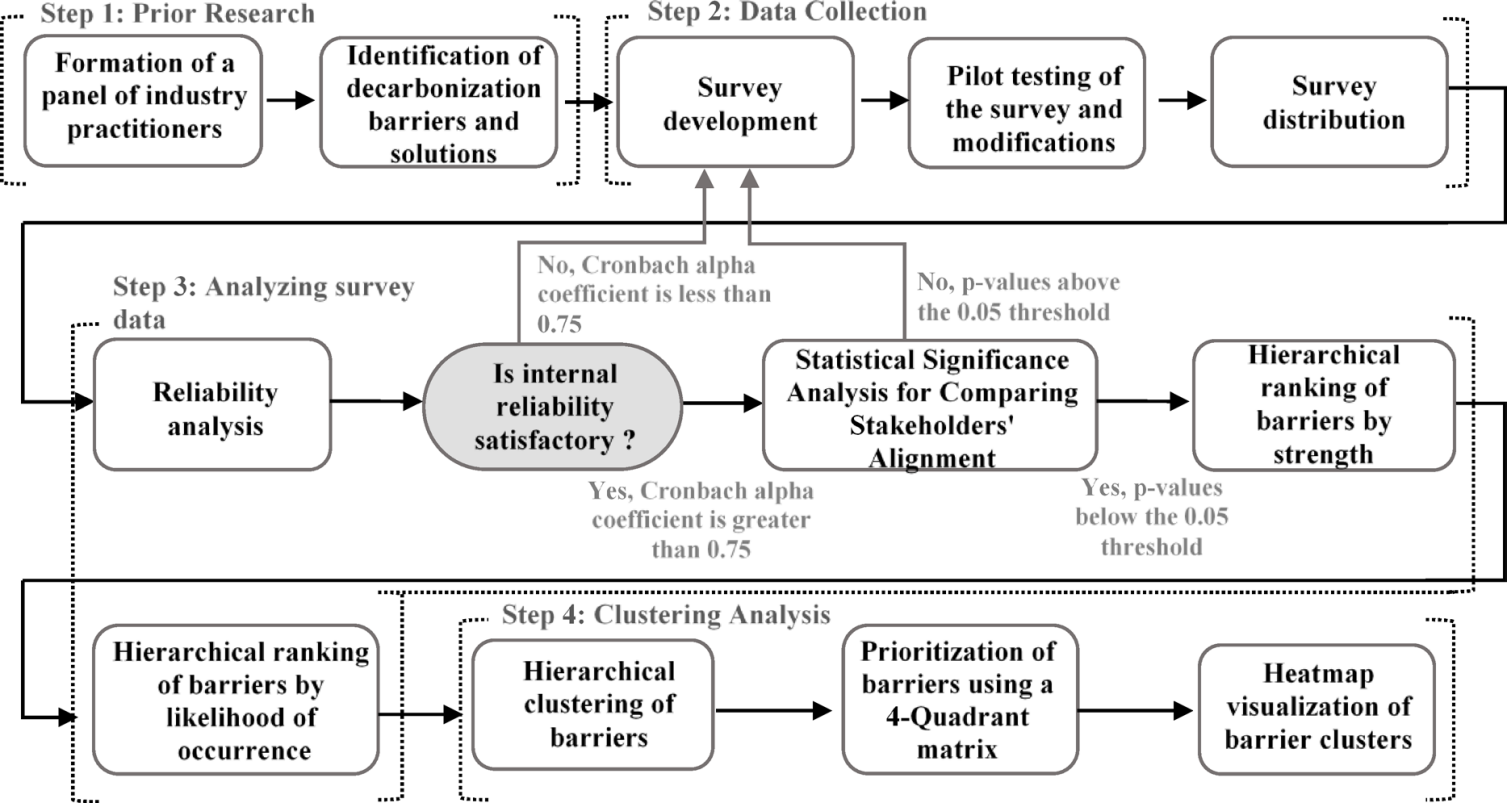
Research methodology.

The research methodology employs a comprehensive four-step approach to systematically identify, evaluate, and prioritize decarbonization barriers in the Egyptian construction sector, as shown in Fig. 1. Step 1 involves a thorough review of prior research to compile an exhaustive list of decarbonization barriers and potential solutions relevant to the construction industry. Step 2 focuses on data collection, beginning with the development of a survey designed to capture stakeholder perspectives. The survey undergoes pilot testing to ensure clarity and relevance, followed by modifications based on feedback before its distribution to a diverse range of industry stakeholders. Step 3 is based on analyzing the collected data to evaluate sample sufficiency and ensure the reliability of the findings, both internally and externally. Statistical significance tests are conducted to compare stakeholder alignment and assess the impact of each barrier using defined evaluation metrics. Finally, Step 4 utilizes advanced clustering techniques to prioritize barriers. This was specifically employed to address the observation that multiple obstacles share similar features, aiming to reveal separate dimensions within the existing set of variables. This includes constructing a four-quadrant matrix to identify high-priority barriers, performing cluster analysis to group barriers based on ratings, and employing heatmap visualizations to enhance the interpretation of barrier clusters. This methodology provides a robust framework for identifying critical challenges and informing actionable solutions for decarbonization in the construction industry.

## Results and analysis

### Step 1: identification of decarbonization barriers and solutions

Through an extensive literature review and input from a panel of 11 industry experts, this study identified: (1) 32 specific barriers to decarbonization in construction projects have been identified; (2) barriers were organized into 6 overarching categories (as outlined in Table 2); and (3) 8 key solutions were identified addressing all the barriers. The industry expert panel included architects, engineers, sustainability consultants, and construction project managers, each with over a decade of professional experience in the construction sector, with a focus on sustainability and decarbonization. Their contributions were instrumental in validating the barriers identified from the literature and in proposing actionable solutions that are context-specific to the challenges of the construction industry.

Barriers and solutions were identified using a dual approach: (1) Literature Review: Provided a foundational understanding of existing challenges and proposed strategies within the global construction industry; and (2) Industry Expert Panel: Offered practical insights, prioritized barriers based on industry experience, and validated the applicability of proposed solutions to real-world contexts. To enhance clarity, Table 3, pairs the numbered barriers with their respective solutions. This visualization is critical as it acts as the core strategic mapping tool of the research, defining which solutions offer the most systemic impact on the identified challenges. For example, the table shows that Solution 6, ‘Drive demand and increase awareness of embodied carbon,’ is the most wide-ranging solution, designed to address eight different barriers, underscoring its importance in tackling a broad range of challenges. Conversely, the concentration of barriers in the ‘Resources’ category, as shown in the table (B1 to B10), indicates that interventions focused on resources are a critical area for action. Furthermore, the explicit linkages in Table 3 provide a clear roadmap for decision-makers by demonstrating, for instance, how Solution 7 (Legislation) is designed to mitigate all four core legislative barriers (B16, B17, B18, B19), plus the technical fear of complexity (B20).

**Table 2:** Summary of identified barriers.

Category	Barrier	
Resources	B1	Low availability of low-carbon materials and equipment
	B2	Limited access to renewable energy sources
	B3	Limited market which prioritizes cost and speed over reducing embodied carbon
	B4	Lack of support from top management
	B5	Difficulty to access good-quality data on embodied carbon
	B6	Inconsistencies between datasets
	B7	Lack of case studies or demonstration projects
	B8	Low availability of skilled labor
	B9	Inadequate training programs
	B10	Inconsistent international standards
Coordination	B11	Lack of supply chain coordination
	B12	Non-involvement during the design and specification stage of the project
	B13	Lack of integration of carbon reduction goals across project phases
	B14	The construction industry involves multiple stakeholders with conflicting interests, making coordinated efforts towards decarbonization difficult.
Legislative	B15	Lack of clear organizational carbon reduction policy
	B16	Lack of clear carbon reduction regulation by the government
	B17	Lack of enforcement mechanisms for existing regulations.
	B18	Insufficient regulatory incentives
	B19	Lack of a single regulating agency for carbon accounting
Technical	B20	Fear of sustainable construction being more complex than conventional construction
	B21	Concerns about durability of low carbon materials
	B22	Insurance issues
	B23	Old and outdated infrastructure may not be easily retrofitted to meet decarbonization goals.
Economic	B24	Perception of higher construction costs
	B25	Difficulty in quantifying carbon reduction benefits
	B26	Anticipated increase in lead times
	B27	Economic fluctuations divert focus from long-term sustainability goals.
	B28	High initial investment costs for low-carbon technologies.
Knowledge and perceptions	B29	Lack of awareness of carbon reduction technologies
	B30	Lack of awareness of the benefits of reducing carbon
	B31	Low priority and other considerations take precedence
	B32	Conservative nature of clients (resistance to embrace new changes)

**Table 3:** Connections between barriers and Solutions.

Solution		Barriers	
S1	Create databases for low-carbon materials, and pilot case projects	B5	Difficulty to access good-quality data on embodied carbon
		B6	Inconsistencies between datasets
		B7	Lack of case studies or demonstration projects
		B10	Inconsistent international standards
S2	Incentives	B23	Old and outdated infrastructure may not be easily retrofitted to meet decarbonization goals.
		B25	Difficulty in quantifying carbon reduction benefits
		B28	High initial investment costs for low-carbon technologies.
		B29	Lack of awareness of carbon reduction technologies
		B32	Conservative nature of clients (resistance to embrace new changes)
S3	Coordination in project phases	B4	Lack of support from top management
		B11	Lack of supply chain coordination
		B12	Non-involvement during the design and specification stage of the project
		B13	Lack of integration of carbon reduction goals across project phases
		B14	The construction industry involves multiple stakeholders with conflicting interests, making coordinated efforts towards decarbonization difficult.
S4	Collaboration between researchers, industry stakeholders, and policymakers	B15	Lack of clear organizational carbon reduction policy
S5	Technology advancement	B1	Low availability of low-carbon materials and equipment
		B2	Limited access to renewable energy sources
		B24	Perception of higher construction costs
S6	Drive demand and increase awareness of embodied carbon	B3	Limited market which prioritizes cost and speed over reducing embodied carbon
		B21	Concerns about durability of low carbon materials
		B22	Insurance issues
		B26	Anticipated increase in lead times
		B27	Economic fluctuations divert focus from long-term sustainability goals.
		B30	Lack of awareness of the benefits of reducing carbon
		B31	Low priority and other considerations take precedence
S7	Legislation	B16	Lack of clear carbon reduction regulation by the government
		B17	Lack of enforcement mechanisms for existing regulations.
		B18	Insufficient regulatory incentives
		B19	Lack of a single regulating agency for carbon accounting
		B20	Fear of sustainable construction being more complex than conventional construction
S8	Training	B8	Low availability of skilled labor
		B9	Inadequate training programs

### Step 2: data collection

#### Survey development

Upon identifying the barriers and solutions influencing decarbonization in the construction industry, an online survey was designed with two primary objectives: (1) to assess the strength (S) of each decarbonization barrier, and (2) to evaluate the likelihood of occurrence (L) for each. A defined Likert scale was provided to guide respondents in rating both S and L. Additionally, the survey gathered respondents’ perspectives on the importance and urgency of eight key solutions for decarbonizing the construction industry in developing countries, such as Egypt. The survey was structured into six sections, each providing clear instructions, definitions, and guidance on how to proceed.

The first section introduced the research study and outlined the objectives of the survey. The second section collected respondent information, including their role in the company, job title, years of experience, and company size. The third section explored respondents’ opinions on who should be primarily responsible for reducing embodied carbon emissions in construction projects. The fourth section assessed participants’ familiarity with various materials and construction products based on their professional experience. To facilitate ease of response, this section was divided into six thematic subsections: (1) Resources; (2) Coordination; (3) Legislative; (4) Technical; (5) Economic; and (6) Knowledge and Perceptions. Within each subsection, respondents were asked to evaluate the criticality and strength of specific barriers related to the theme. The fifth section, also divided into the same six subsections, focused on assessing the likelihood of occurrence for each identified barrier.

A 5-point Likert scale, shown in Table 4 were utilized with clear descriptions provided at the start of each section to ensure respondents understood the meaning of the scale. The final section in the survey used a similar 5-point Likert scale to evaluate the perceived importance and urgency of proposed solutions for decarbonizing the construction industry. Detailed explanations of the scale were included to ensure consistency in responses. This section aimed to prioritize key actions needed to advance decarbonization efforts, particularly in the context of developing countries.

**Table 4:** Adapted scale for assessing barriers and Solutions.

Likert scale	Barriers		Solutions
	Barrier Strength	Likelihood of Occurrence	
NA	-	-	**Not Applicable**: This action is irrelevant to the decarbonization efforts in the construction industry
1	**Very Weak**: The barrier has minimal or no impact on decarbonization efforts.	**Very Low Probability**: The barrier is not important and can be deprioritized, as it does not require immediate or significant attention.	**Not Important**,** Not Urgent.** The action has a low impact on decarbonization and can be deprioritized. It does not require immediate or significant attention
2	**Weak**: The barrier exists but has a relatively low impact on decarbonization efforts.	**Low Chance**: The barrier is unlikely to occur, with a small chance (11–30%), making it a lower priority concern.	**Slightly Important**,** Slightly Urgent**: The action has a moderate impact and should be considered, but it does not demand urgent attention. Its implementation can be flexible
3	**Moderate**: The barrier is noticeable, having a moderate impact on decarbonization efforts	**Medium Chance**: The barrier has a moderate likelihood of occurring (31–65%). It requires attention but is not a critical obstacle.	**Important**,** Not Urgent**: The action is essential for decarbonization but does not require immediate attention. It can be implemented gradually without major negative consequences
4	**Strong**: The barrier has a substantial impact, significantly hindering decarbonization efforts.	**High Chance**: The barrier is likely to occur (66–90%). It demands focused efforts to address.	**Important**,** Urgent**: The action is critical and needs to be addressed immediately to achieve decarbonization. Delaying will significantly hinder progress
5	**Very Strong**: The barrier is highly influential, severely obstructing decarbonization progress.	**Vert High Chance**: The barrier is almost certain to occur (90% or greater). It requires immediate and strategic action to overcome.	**-**

#### Pilot testing of the survey and modifications

The expert-based survey underwent a comprehensive review by the authors and research collaborators before being pilot-tested to ensure its effectiveness and address potential issues. At the conclusion of the pilot phase, participants were invited to provide detailed feedback on various aspects, such as identifying additional decarbonization barriers or solutions that could be added, modified, or removed. A total of 10 experts suggested adjustments to existing questions, offered clarifications to enhance consistent understanding, and provided general recommendations to improve the survey’s quality and precision. This valuable feedback was carefully recorded and thoroughly analyzed to refine the survey, ensuring it was both robust and aligned with the research’s objectives.

#### Survey distribution

An open online questionnaire platform was selected as the most effective format, enabling practitioners to easily share the survey and ensuring broad accessibility to maximize the number and diversity of responses. The survey link was distributed via multiple channels, including specialized online groups, LinkedIn networks, and directly to a targeted group of individuals with significant expertise in construction and sustainability. To ensure the collection of relevant insights, selective sampling was utilized to distribute the finalized survey to 146 practitioners within Egypt’s construction and sustainability sectors. Of these, 146 participants responded, but 21 submissions were incomplete or insufficient. As a result, 125 valid responses were included in the analysis.

This study was reviewed and approved by the Institutional Review Board of The American University in Cairo (IRB Case #2024-2025-168). All research procedures were conducted in strict accordance with the IRB’s ethical guidelines for human subjects research, with particular emphasis on participant protection, data confidentiality, and anonymity safeguards.

Prior to accessing the survey, all participants were required to complete an informed consent process that clearly outlined: (1) the study purpose and objectives, (2) research procedures, (3) expected duration, (4) potential risks and benefits, and (5) data protection measures. Written informed consent was obtained from all 146 participating construction and sustainability sector practitioners in Egypt, confirming their voluntary participation based on full understanding of the study parameters.

### Step 3: analyzing survey data

#### Sample sufficiency

To evaluate whether the sample size accurately represents the current state of the industry, statistical methods are applied using Eq. 1. These methods are commonly used in statistical analysis to determine the minimum sample size required to reliably estimate the population mean. In this equation, ***n*** represents the minimum sample size, ***z*** is the standard normal deviation (with ***z*** = 1.96 for a 95% confidence level), ***d*** is the acceptable standard error of the mean, and ***s*** is the population standard deviation. It is assumed that the sample standard deviation is equivalent to the population standard deviation, a reasonable assumption often made by statisticians when the population standard deviation is unknown.

1 $$\:\boldsymbol{n}=\:\frac{{\boldsymbol{z}}^{2}{\boldsymbol{s}}^{2}}{{\boldsymbol{d}}^{2}}$$

**Table 5:** Sample size analysis.

Standard normal deviation (z)	Population standard deviation (s)	Acceptable standard error of mean (d)	Sample size (*n*)
1.96	1.96	12.5% = 0.5	60
1.96	1.96	10% = 0.4	92
1.96	1.96	8.5% = 0.35	125
1.96	1.96	7.5% = 0.3	164
1.96	1.96	6.25% = 0.25	236
1.96	1.96	5% = 0.2	369

Based on Table 5 sample size of 92 is required for a standard error of 10%. Since the expert-based survey exceeds this with 33 responses, ***the sample size can be considered sufficient.*** If a standard error of 7.5% were desired, the required sample size would increase to 164. However, a standard error of 8.5% is deemed acceptable in this case.

#### Characteristics of surveyed professionals

**Fig. 2:**
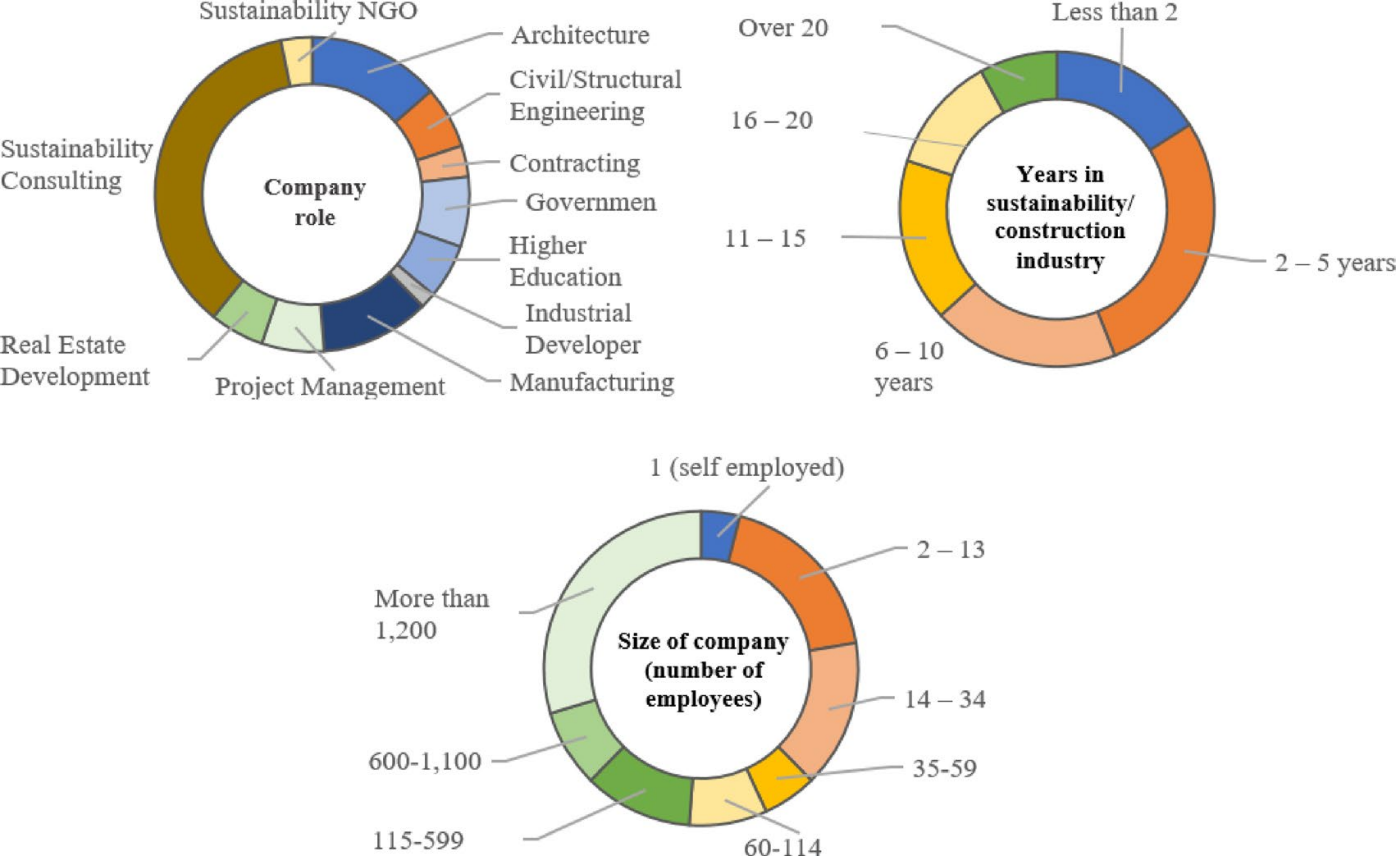
Respondent demographics.

The total number of expert respondents with complete responses is 125. The following section examines the adequacy of the sample size using statistical methods to ensure that the data obtained from these 125 experts are reliable and representative of the industry. These practitioners possess substantial experience in the construction sector in Egypt, as well as in environmental consulting. This extensive experience ensures that their responses are informed and well-rounded, making them representative of the construction industry in Egypt. The distribution of surveyed industry practitioners across various company roles is illustrated in Fig. 2 with stakeholders further categorized into groups based on their roles, as detailed in Table 6.

Figure 2 provides an overview of the respondent demographics, highlighting that the majority were sustainable consultants and architects, followed by professionals from the manufacturing sector. Most respondents had 11–15 years of experience indicating a pool of participants with substantial expertise in the field. Additionally, many were employed by large organizations with over 1,200 employees, underscoring their involvement in significant industry operations.

**Table 6:** Survey stakeholder groups and roles.

Group	Stakeholders
Design and Development	Architecture Real estate developer Industrial developer
Sustainability & Compliance	Sustainability consultant Sustainability NGO
Compliance	Government or Regulatory body
Engineering & Execution	Civil/structural engineer Project management
Manufacturing & Supply chain	Manufacturing

#### Reliability analysis

A series of questions were included in the survey, and Cronbach’s Alpha was used to assess the internal reliability of the responses provided by each respondent, shown in Table 7. Cronbach’s Alpha assesses how closely related a set of items (questions or indicators) are as a group. An alpha value closer to 1.0 indicates high reliability, whereas values below 0.7 may suggest a lack of consistency. High reliability is seen in the evaluation of barriers (α = 0.94) and the likelihood of barriers (α = 0.99), indicating that the measures used for these topics are well-developed and provide highly consistent results. Moderate reliability (α = 0.73) is observed in the ranking of stakeholders, which suggests that while the measure is reliable, there may be some inconsistency in how stakeholders’ roles are perceived or ranked. Excellent reliability (α = 0.95) is observed in the evaluation of decarbonization actions, highlighting that the importance of strategies is consistently recognized across respondents.

**Table 7:** Internal reliability analysis.

	Cronbach’s Alpha (α)
Ranking stakeholders’ roles in minimizing embodied carbon emissions on construction projects	0.73
Evaluating the strength of barriers in a set of 32 identified challenges	0.94
Assessing the likelihood of occurrence of barriers from a set of 32 challenges	0.99
Evaluating the importance of actions for decarbonizing the construction industry in developing countries, such as Egypt, from a list of 8 key strategies	0.95

#### Statistical significance analysis for comparing stakeholders’ alignment

To understand stakeholder perspectives on who should bear responsibility for minimizing embodied carbon emissions in construction projects, respondents were asked to rank eight key stakeholder groups in terms of their responsibility: (1) Architects, (2) Clients, (3) Sustainability Consultants, (4) Civil/Structural engineers, (5) Contractors, (6) Project Management Offices, (7) Governmental or Regulatory Bodies, and (8) Sustainability NGOs. A Chi-square test was used to determine whether the observed rankings significantly diverged from an expected uniform distribution. All p-values are well below the threshold of 0.05, indicating strong consensus among respondents regarding the distribution of responsibility. The likelihood that these results occurred by chance is negligible. The lowest p-values were observed for Governmental or Regulatory Bodies and Sustainability Consultants. This suggests a nearly unanimous agreement that these two groups hold the greatest responsibility for addressing embodied carbon emissions. Architects, civil/structural engineers, and contractors were also identified as significant contributors, reflecting the operational and design responsibilities inherent to their roles. Clients, project management offices (2.7E-09), and sustainability NGOs were ranked with slightly higher p-values but still show significant divergence from a uniform ranking. This indicates recognition of their roles, though with comparatively less consensus.

The results highlight the multi-layered nature of responsibility for minimizing embodied carbon emissions, emphasizing the need for collaboration between technical, managerial, and regulatory stakeholders. The consensus on the role of governmental bodies underscores the importance of implementing robust regulations and incentives to drive decarbonization in the construction sector. Similarly, the significance attributed to sustainability consultants reflects the value of expertise in carbon reduction strategies, suggesting that increased investment in training and consultancy could enhance industry outcomes.

In analyzing the P-values from the Chi-square test for the 8 solutions to decarbonization and addressing barriers, we observe that all the solutions demonstrate highly statistically significant results, with P-values well below the typical threshold of 0.05 (Table 8). This indicates a strong alignment among the stakeholders regarding the importance of these solutions in decarbonization efforts. The solutions that garnered the most significant support from stakeholders include Incentives, Legislation, and Collaboration between researchers, industry stakeholders, and policymakers. These solutions, with extremely low P-values, reflect near-unanimous agreement that financial incentives, robust legislation, and cross-sector collaboration are vital for advancing decarbonization. Stakeholders clearly recognize that such policy-driven solutions are essential for creating the framework necessary to achieve meaningful progress. While all solutions demonstrate strong consensus, “Drive demand and increase awareness of embodied carbon” exhibits a slightly higher P-value compared to others, though it remains highly statistically significant. This indicates that stakeholders widely recognize the importance of raising awareness and fostering demand for low-carbon solutions. However, there is marginally less agreement on its immediate priority compared to more direct measures such as incentives and legislation. Despite this, the findings underscore substantial support for integrating awareness-building and demand-generation initiatives into comprehensive decarbonization strategies.

**Table 8:** P-Values from Chi-Square test for Solutions.

Solutions		*P*-Value using Chi-square test
S1	Create databases for low-carbon materials, and pilot case projects	7.5E-14
S2	Incentives	1.8E-18
S3	Coordination in project phases	2.0E-15
S4	Collaboration between researchers, industry stakeholders, and policymakers	3.9E-15
S5	Technology advancement	2.5E-14
S6	Drive demand and increase awareness of embodied carbon	1.4E-09
S7	Legislation	2.1E-17
S8	Training	8.2E-12

#### Barrier evaluation metrics

Table 9 provides a comprehensive evaluation of 32 identified barriers, presenting their mean, standard deviation, and ranking based on both the strength and likelihood of occurrence for each barrier. Barrier ratings were determined by multiplying the mean strength by the mean likelihood of occurrence for each barrier. This quantification facilitated a deeper understanding, prioritization, and ranking of the critical barrier factors impacting decarbonization in construction projects. The barrier rating *(BR*_*i*_*)* was calculated using the following formula in Eq. 2:

2 $$\:{BR}_{i}\frac{1}{N\:}\sum_{r=1}^{N}{S}_{ri}{L}_{ri\:\:}$$

The barrier rating (*BR*_*i*_​) calculation, determined by multiplying the mean strength (*S*) by the mean likelihood (*L*) of occurrence, serves a vital statistical purpose: it creates a prioritization metric that quantifies the combined risk of a barrier’s impact and its frequency. This calculation, based on responses from all 125 surveyed practitioners (*N* = 125), produces a single metric that prioritizes barriers by quantifying their combined impact and probability, ensuring that intervention efforts are directed toward factors that are both powerful and probable. The statistical significance of these ratings is confirmed by the P-value. Barriers with extremely low P-values for both strength and likelihood (such as B03, B24, and B28) signify strong consensus among experts regarding their significant impact and frequent occurrence, marking them as critical areas of concern that warrant immediate attention. Conversely, barriers exhibiting substantial variability between strength and likelihood (e.g., B02) warrant further investigation to better understand the differences in stakeholder perceptions.

The p-values for the strength of barriers reflect the perceived impact these challenges have on projects. Barriers such as B03 (3.9E-13), B16 (1.9E-13), and B24 (1.1E-16) show extremely low p-values (< 0.01), indicating strong stakeholder consensus regarding their high impact. These barriers are viewed as critical challenges that must be addressed to facilitate decarbonization. Moderately significant barriers, with p-values between 0.01 and 0.05, such as B06 (2.7E-02) and B09 (2.3E-02), exhibit moderate significance, suggesting some variability in stakeholder perceptions. While these barriers are impactful, they may not be universally recognized as primary concerns. Barriers B08 (1.6E-01) and B10 (2.0E-01) are the only ones with p-values above the 0.05 threshold, indicating lower levels of agreement on their influence. These results suggest that stakeholders perceive these barriers as less critical, or there may be inconsistencies in their understanding of these barriers’ impact.

The p-values for likelihood of occurrence represent stakeholders’ consensus on the frequency or probability of these barriers arising in practice. Barriers like B03 (9.8E-13), B24 (3.9E-15), and B28 (7.0E-12) demonstrate very low p-values, highlighting strong agreement among stakeholders regarding their frequent occurrence. These barriers represent persistent challenges that projects are likely to encounter. Barriers such as B06 (9.2E-03) and B07 (1.1E-02) indicate moderate consensus on their likelihood. These barriers may arise periodically, depending on project-specific factors. Barriers B08 (1.6E-03) and B10 (3.3E-03) are less frequently perceived as probable challenges, with p-value above the threshold level. These results suggest variability in stakeholders’ experiences or expectations related to these barriers. Barriers B03, B24, and B28 have highly significant p-values for both dimensions, marking them as critical areas of concern. These barriers warrant immediate attention, as they pose substantial challenges and occur frequently. Barriers B02 and B10 show differences between strength and likelihood. For instance, B02 has a moderately significant strength p-value (4.8E-02) but a highly significant likelihood p-value (2.8E-04), suggesting that while stakeholders may agree on its frequent occurrence, they may differ on its perceived impact.

These findings offer a data-driven basis for effective resource prioritization, enabling policymakers and project teams to focus on the most critical barriers. Barriers with low p-values for both strength and likelihood should be prioritized for intervention, as they signify both significant impact and frequent occurrence. Conversely, barriers exhibiting substantial variability between strength and likelihood warrant further investigation to better understand the differences in stakeholder perceptions and to refine strategies for addressing these challenges.

**Table 9:** Results for strength, likelihood of occurrence, barrier rating and P-values.

Category	Barrier	Strength (S)			Likelihood of occurrence (L)			Barrier Rating (BR)	P-Value using T-test	
		Mean (1–5)	Std dev	Rank	Mean (1–5)	Std dev	Rank		Strength of the barriers	Likelihood of occurrence
Resources	B1	3.66	1.25	18	3.64	1.29	19	13.28	0.15	0.77
	B2	3.38	1.27	22	3.43	1.25	25	11.49	0.59	0.79
	B3	4.06	1.17	3	4.07	1.08	2	16.49	0.46	0.59
	B4	3.83	1.06	13	3.72	1.19	14	14.90	0.47	0.49
	B5	3.59	1.23	19	3.46	1.21	24	12.42	0.77	0.44
	B6	3.21	1.28	28	3.34	1.22	29	10.72	0.64	0.77
	B7	3.18	1.18	30	3.33	1.24	28	10.57	0.58	0.58
	B8	3.36	1.35	26	3.34	1.19	27	11.21	0.39	0.98
	B9	3.23	1.22	27	3.31	1.19	29	10.71	0.68	0.44
	B10	3.12	1.28	31	3.18	1.21	32	9.98	0.09	0.40
Coordination	B11	3.22	1.12	29	3.43	1.10	26	11.03	0.84	0.12
	B12	3.79	1.12	14	3.72	1.07	14	14.25	0.24	0.19
	B13	4.00	1.14	5	3.75	1.19	12	14.98	0.63	0.26
	B14	3.88	1.14	11	3.91	1.20	4	15.17	0.52	0.41
Legislative	B15	3.91	1.09	8	3.75	1.18	14	14.42	0.38	0.35
	B16	4.08	1.11	2	3.88	1.20	7	15.82	0.86	0.65
	B17	4.02	1.16	4	3.86	1.14	8	15.51	0.41	0.96
	B18	3.93	1.06	7	3.93	1.14	5	15.50	0.07	0.93
	B19	3.66	1.16	17	3.63	1.21	20	13.68	0.39	0.44
Technical	B20	3.58	1.21	21	3.82	1.18	10	13.32	0.14	0.37
	B21	3.38	1.15	24	3.64	1.18	18	12.35	0.17	0.11
	B22	3.04	1.09	32	3.29	1.25	31	9.94	0.81	0.74
	B23	3.42	1.13	23	3.61	1.16	22	12.29	0.16	0.48
Economic	B24	4.17	1.06	1	4.16	1.08	1	17.36	0.50	0.72
	B25	3.61	1.22	20	3.57	1.25	20	12.88	0.37	0.45
	B26	3.31	1.04	25	3.47	1.18	23	11.62	0.94	0.40
	B27	3.96	1.05	9	3.92	1.11	6	15.42	1.00	0.86
	B28	4.06	1.05	6	4.06	1.14	3	16.46	0.62	0.54
Knowledge and perceptions	B29	3.85	1.22	12	3.73	1.23	13	14.10	0.08	0.01
	B30	3.73	1.24	16	3.66	1.23	17	13.68	0.76	0.01
	B31	3.80	1.17	15	3.80	1.13	9	14.34	0.34	0.40
	B32	3.93	1.15	10	3.79	1.18	11	14.67	0.94	0.99

Table 10 provides an analysis of the perceived responsibility of different stakeholder groups in decarbonization efforts, reflecting their ranking, mean scores, and standard deviations. The Governmental or Regulatory Body ranks highest, with a mean score of 4.48, indicating strong consensus among respondents about its critical role in driving decarbonization. This group has the lowest standard deviation, suggesting broad agreement on its importance. Following closely, the Sustainability Consultant ranks second with a mean score of 4.42. While their responsibility is seen as almost as significant as the governmental body, the slightly higher standard deviation (0.88) reflects a bit more variation in opinions. Contractors are ranked eighth with the lowest mean score of 3.55. The moderate standard deviation (0.96) suggests that while contractors are seen as playing a role in decarbonization, they are considered less responsible compared to other stakeholder groups.

Overall, the results suggest that governmental bodies and sustainability consultants are seen as the most responsible for driving decarbonization, with varying levels of agreement about the roles of other stakeholders. A two-tailed T-test was conducted to calculate the p-value for both the strength of barriers and their likelihood of occurrence, revealing no statistically significant difference in perceptions between sustainability consultants and other respondents. This indicates a general alignment in how these two groups view the barriers, suggesting that their assessments of both the intensity and probability of these barriers are consistent.

**Table 10:** Evaluation metrics of stakeholder responsibility in decarbonization Efforts.

Stakeholder	Mean	Std dev	Rank
Architect	3.86	1.05	3
Client	3.70	1.16	4
Sustainability Consultant	4.42	0.88	2
Civil/Structural engineer	3.58	0.99	7
Contractor	3.55	0.96	8
Project Management Office	3.66	1.02	6
Governmental or Regulatory Body	4.48	0.78	1
Sustainability NGOs	3.68	1.31	5

Table 11 presents an analysis of seven potential solutions for addressing decarbonization challenges in construction, ranking them based on their perceived effectiveness, with corresponding mean scores, standard deviations (std dev), and ranks. The solution ranked first is Incentives (mean: 3.41, std dev: 0.83). This suggests that respondents view incentives as the most effective solution to drive decarbonization efforts. The relatively moderate standard deviation indicates some variability in opinions, but the solution is generally regarded as important. Legislation ranks second (mean: 3.37, std dev: 0.87), with respondents acknowledging its crucial role in supporting decarbonization efforts. The standard deviation indicates some degree of disagreement, but overall, respondents believe that legal frameworks and regulations will play a significant part in advancing decarbonization. Drive demand and increase awareness of embodied carbon ranks seventh (mean: 3.19, std dev: 0.93), indicating that while it is recognized as a necessary step, it is considered less impactful than other solutions. The higher standard deviation suggests a greater divergence of opinion on its effectiveness. Overall, the analysis shows that respondents prioritize incentives and legislation as the most crucial solutions, with technology advancement and creating databases for low-carbon materials receiving less emphasis. The relatively high standard deviations for most solutions indicate some level of disagreement on the exact approaches to take for effective decarbonization in the construction sector.

**Table 11:** Evaluation metrics for solutions for decarbonization in the construction Sector.

Solution		Mean (1–5)	Std dev	Rank
S1	Create databases for low-carbon materials, and pilot case projects	3.30	0.81	5
S2	Incentives	3.41	0.83	1
S3	Coordination in project phases	3.36	0.81	3
S4	Collaboration between researchers, industry stakeholders, and policymakers	3.35	0.82	4
S5	Technology advancement	3.30	0.73	5
S6	Drive demand and increase awareness of embodied carbon	3.19	0.93	7
S7	Legislation	3.37	0.87	2

### Step 4: clustering analysis

#### Prioritization of barriers using a 4-quadrant matrix

**Fig. 3:**
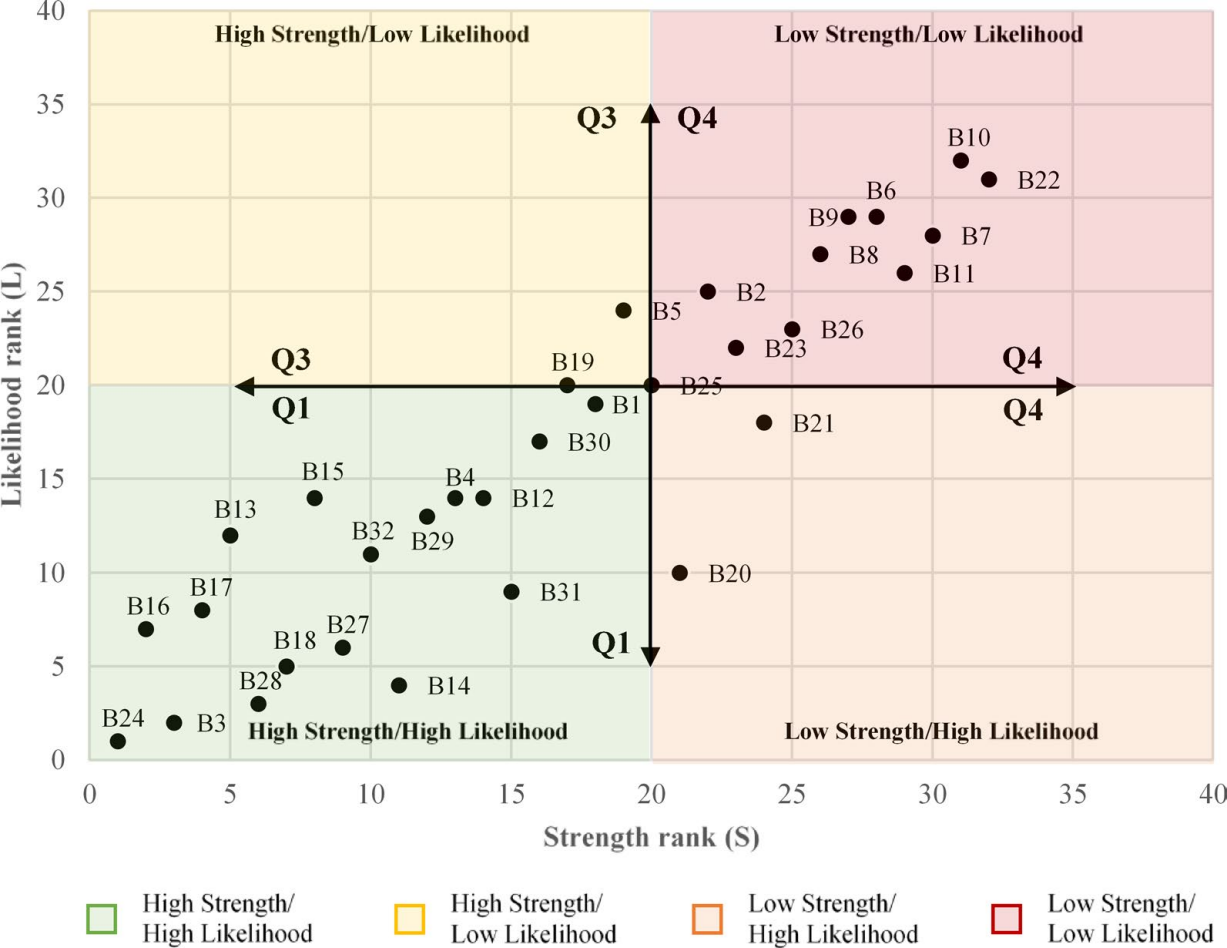
Priority Matrix: Strength vs. Likelihood of Occurrence.

The 4-quadrant priority matrix (Fig. 3) provides a valuable framework for identifying and prioritizing barriers based on their strength and likelihood of occurrence. The matrix categorizes the barriers into four quadrants: Q1 (High Strength / High Likelihood), Q2 (Low Strength / High Likelihood), Q3 (High Strength / Low Likelihood), and Q4 (Low Strength / Low Likelihood), each representing different levels of urgency and impact. Q1 (High Strength / High Likelihood) is the most critical quadrant, containing 18 barriers. These barriers are both highly impactful and very likely to occur, making them the top priority for immediate action.

The perception of higher construction costs (B24) stands out in this quadrant, ranking first in both strength and likelihood. This barrier reflects widespread concerns in the industry regarding the financial feasibility of low-carbon construction, making it the highest-ranked and most urgent issue to address. Alongside B24, regulatory issues (B16, B17, B18) and market dynamics (B3, B28) emerge as prominent high-priority barriers, suggesting a need for systemic reforms and financial incentives to overcome these obstacles. Q2 (Low Strength / High Likelihood) includes 3 barriers that are likely to occur but have lower immediate impact. These barriers, while still significant, may not demand as urgent intervention as those in Q1, but they warrant strategic consideration to mitigate their future effects. Q3 (High Strength / Low Likelihood) features 3 barriers that are highly impactful but less likely to happen. These barriers require proactive monitoring and contingency planning to prepare for potential future challenges, though their lower likelihood means they may not demand immediate resources. Q4 (Low Strength / Low Likelihood) is the least critical quadrant, consisting of 11 barriers with low rankings in both strength and likelihood. These barriers are not expected to have significant immediate effects and can be addressed later or as resources permit.

#### Results of k-means clustering

**Fig. 4:**
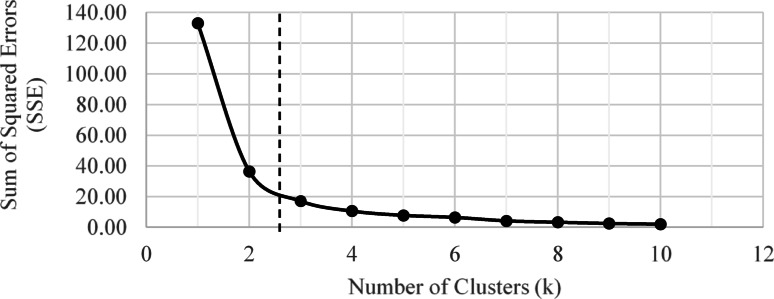
Elbow Plot.

Clustering analysis was employed to systematically group the identified factors into categories of varying levels of criticality based on survey respondents’ ratings. To determine the optimal number of clusters (k) for the algorithm, an elbow plot (Fig. 4) was generated. This plot visually depicts the relationship between the number of clusters and the data’s distortion, measured as the sum of squared errors (SSE) between data points and their respective cluster centroids. The “elbow” point, where the reduction in distortion begins to taper off, indicated that three clusters provided the most appropriate grouping.

**Fig. 5:**
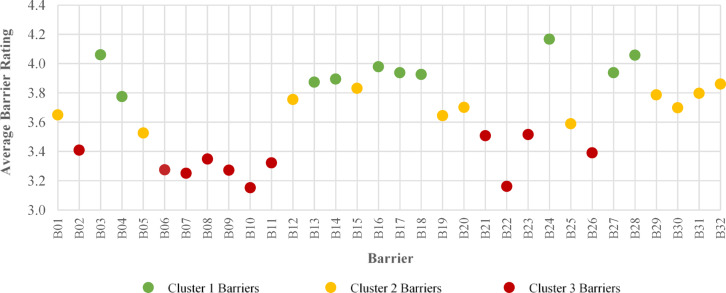
Clustered decarbonization barriers and their ratings.

After identifying the optimal number of clusters, the k-means algorithm was applied to analyze the ratings of 32 decarbonization barriers. The results, presented in Table 12; Fig. 5 categorized the barriers into three distinct clusters, each reflecting varying levels of criticality and comprising a unique set of factors. All barriers included in the analysis had ratings of 9.94 or higher.

The grouping of barriers into three distinct clusters provides valuable thematic insights into their interconnected nature and criticality.

- Cluster 1 (Most Critical), encompassing 10 barriers, is defined by fundamental systemic issues requiring immediate action. The clustering of core Legislative factors (B16, B17, B18), including the lack of clear regulation and enforcement mechanisms, with major Economic barriers (B24, B27, B28) infers that regulatory voids and financial constraints are intrinsically linked and represent the primary, most urgent inhibitors to decarbonization progress.
- Cluster 2 (Significant) includes barriers where organizational change and data gaps are prominent. The presence of barriers like B15 (Lack of clear organizational policy) alongside B05 (Difficulty to access good-quality data on embodied carbon) suggests these are mid-level challenges where internal institutional will and specific expertise/data gaps must be addressed to advance.
- Cluster 3 (Least Critical), consisting of 11 barriers, includes residual technical and general resource limitations (e.g., B02, B21, B22, B23) that can be addressed over a longer timeframe. These barriers, while notable, are considered the least critical and can be addressed over a longer timeframe. By dividing the barriers according to their criticality, the clustering algorithm provided valuable insights, allowing for the identification of groups with varying degrees of urgency.

This analysis serves as a foundation for prioritizing mitigation efforts and formulating targeted recommendations to address decarbonization challenges effectively.

**Table 12:** Cluster analysis of decarbonization barriers based on barrier Ratings.

Category	Cluster 1	Cluster 2	Cluster 3
Resources-related barriers	B03 - B04	B01 - B05	B02 - B06 - B07 - B08 - B09 - B10
Coordination-related barriers	B13 - B14	B12	B11
Legislative-related barriers	B16 - B17 - B18	B15 - B19	–
Technical-related barriers	–	B20	B21 - B22 - B23
Economic-related barriers	B24 - B27 - B28	B25	B26
Knowledge and perception-related barriers	–	B29 - B30 - B31 - B32	–

**Fig. 6:**
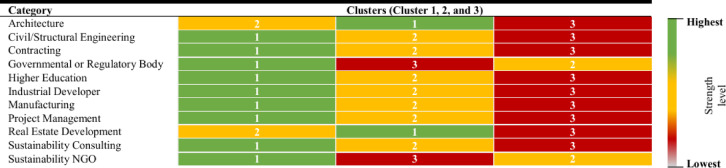
Heatmap visualization of strength across clusters.

**Fig. 7:**
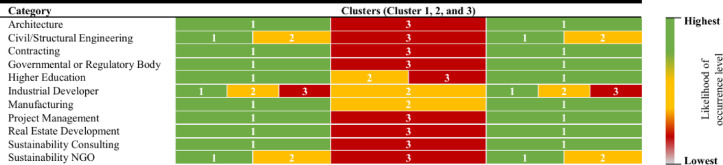
Heatmap visualization of likelihood of occurrence across clusters.

The heat map visualizations based on respondents’ company roles, examines their perception of the strength of decarbonization barriers, grouped into three clusters (Figs. 6 and 7). This approach offers valuable insights into how different sectors prioritize these challenges, allowing for a more targeted and strategic response. As illustrated in Fig. 6, all sectors, except Architecture and Real Estate Development, consider the barriers in Cluster 1 the most urgent and critical, demanding immediate attention. In contrast, professionals in Architecture and Real Estate Development perceive the barriers in Cluster 2 as the most pressing in terms of their strength. Cluster 3, across all sectors, is consistently viewed as the least critical, with its barriers seen as less immediate and more suited for long-term resolution. When considering the likelihood of occurrence, several clusters show similar rankings, suggesting that the probability of encountering these barriers is not confined to one cluster. Notably, Cluster 3 is commonly perceived at a moderate level, indicating that while these barriers are significant, they are not considered as urgent as those in the higher-priority clusters.

### Analysis of findings

Understanding the various factors influencing decarbonization in construction projects is crucial for the industry, enabling progress across multiple dimensions. First, it provides stakeholders, including contractors, engineers, policymakers, and clients, with actionable insights to prioritize critical barriers and address them systematically. This prioritization supports the development of efficient mitigation strategies that align with project goals while minimizing environmental impact. Additionally, the clustering of barriers based on criticality offers a structured approach to resource allocation, ensuring that immediate efforts are directed toward the most pressing challenges while less urgent issues are planned for long-term resolution. This structured framework not only enhances decision-making but also facilitates more effective collaboration among stakeholders by clarifying roles and responsibilities in tackling decarbonization challenges.

Furthermore, these insights play a pivotal role in shaping policy development by highlighting systemic gaps and uncovering opportunities for regulatory and institutional support. For instance, legislative barriers such as B16, B17, and B18 identified in Cluster 1, alongside technical barriers like B20 in Cluster 2, underscore the need for more comprehensive regulations and targeted incentives. Addressing these barriers can lead to the establishment of industry standards and financial mechanisms that encourage stakeholders to adopt low-carbon practices and technologies, ultimately advancing decarbonization efforts. From an academic perspective, these findings enrich the knowledge base surrounding sustainable construction, offering a robust foundation for future research into innovative solutions, low-carbon materials, and advanced construction technologies. By identifying critical barriers, this study provides a roadmap for exploring uncharted areas of decarbonization, fostering cross-disciplinary collaborations to tackle these challenges. The highest-ranking barrier in terms of criticality, B24, which pertains to the perception of higher costs, along with the second-ranked barrier, B3, which relates to a limited market that prioritizes cost and speed over reducing embodied carbon, highlights fundamental market dynamics that must shift to enable meaningful progress. These findings suggest that addressing cost-related perceptions through awareness campaigns, financial incentives, and showcasing successful low-carbon projects could help drive broader adoption of sustainable practices. Moreover, encouraging market demand for low-carbon solutions by integrating embodied carbon reduction as a key performance indicator in construction projects can accelerate the transition toward sustainability.

The study’s findings align with barriers observed in other developing economies. For example, the high prevalence of economic barriers (B24, B28) and low awareness (B30) corroborates research in similar regional contexts, such as the UAE and Kuwait, where lack of awareness was identified as a primary obstacle to implementing sustainable construction. This suggests that the fundamental challenges of cost perception and market immaturity are shared across the Gulf region and Egypt. The findings of this study both support and expand upon previous studies. The critical ranking of perception of higher construction costs (B24) and high initial investment costs (B28) corroborates prior research^4,5^ establishing cost as the primary universal challenge that impedes decarbonization globally. Furthermore, the high priority assigned to legislative barriers (B16, B17, B18) supports the argument by Jaglan and Korde^8^ regarding the necessity of government-enforced climate legislation to drive systemic change. Crucially, this research provides novel, context-specific implications for the Egyptian market. The high ranking of economic and data barriers (B24, B28, B5), which includes difficulty accessing good-quality data on embodied carbon, demonstrates that these challenges “prevent stakeholders from effectively evaluating OC-EC trade-offs”. This explains why the optimal adoption of Whole-Life Carbon (WLC) analysis, a core concept acknowledged in global sustainability frameworks, is fundamentally impeded in the Egyptian market by the identified local barriers. The high priority of legislative barriers also reflects the specific local void where the Egyptian Code for Improving the Efficiency of Energy Use in Buildings (ECP 306–2017) focuses solely on operational energy and does not address embodied carbon.

The barriers identified in this study, particularly the economic ones (B24, B28) and those related to data and knowledge (B5, B29, B30), have direct implications for the adoption of a whole-life carbon approach. The high perceived cost of low-carbon investments and the lack of robust carbon data prevent stakeholders from effectively evaluating OC-EC trade-offs. For instance, a developer facing barrier B24 (perception of higher construction costs) is unlikely to invest in a high-performance envelope that increases EC but significantly reduces OC, as the long-term operational savings cannot be justified against the upfront cost within the current market context. Similarly, without solutions to barrier B5 (difficulty to access good-quality data), practitioners cannot accurately model these trade-offs. Therefore, the solutions proposed in this paper, such as legislation (S7), incentives (S2), and creating databases (S1) are not just for reducing EC in isolation; they are essential enablers for creating a market and a knowledge base where informed, optimal WLC decisions can become standard practice.

While ranking stakeholder responsibility is valuable, a deeper analysis of our data reveals underlying power imbalances and potential conflicts of interest that could significantly hinder collaborative decarbonization efforts. The significant variance in how different groups perceive both barriers and responsibility (as shown in Table 10; Figs. 6 and 7) is not merely a difference of opinion but a symptom of deeper structural challenges.

1. **Power Imbalance and Implementation Resistance**: A clear power imbalance is evident in the responsibility rankings (Table 10). Stakeholders overwhelmingly assign primary responsibility to Governmental or Regulatory Bodies (Rank 1), who hold the power to mandate and enforce change. In sharp contrast, Contractors and Civil Engineers (Ranks 7 & 8), who are directly responsible for material selection and construction execution, are assigned the least responsibility. This suggests a dynamic where on-the-ground actors may view decarbonization as a constraint *imposed upon them* by powerful external regulators, which can lead to resistance in the form of minimal compliance or a reluctance to innovate beyond the letter of the law.
2. **Fundamental Conflicts of Interest**: The heatmap analysis (Figs. 6 and 7) reveals fundamental conflicts of interest and priority. For instance, Architects and Real Estate Developers perceive the barriers in Cluster 2 (which includes economic and market barriers) as most critical. Conversely, all other sectors, including Sustainability Consultants and Government, prioritize the legislative and coordination barriers in Cluster 1 as most pressing. This divergence highlights a core conflict: designers and developers prioritize the financial viability and marketability of projects, while other groups focus on systemic and regulatory change. Without mechanisms to align these interests, this conflict will result in a stalemate.
3. **Institutional Resistance**: The data suggests institutional resistance to change. The low responsibility assigned to contractors and engineers, coupled with their distinct perspective on barrier criticality, points to a sector that may be resistant to absorbing new risks and costs associated with low-carbon practices. Furthermore, the high ranking of “Lack of support from top management” (B4) and “Lack of clear organizational carbon reduction policy” (B15) indicates resistance within private organizations themselves, where short-term profit motives often override long-term sustainability goals unless mandated or heavily incentivized.

Therefore, overcoming decarbonization barriers is not solely a technical or regulatory challenge but a socio-political one. Effective strategies must acknowledge and manage these dynamics: regulations from high-power entities (Government) must be designed to empower, not just mandate, low-power but high-implementation actors (Contractors). The findings of this study, particularly the high ranking of legislative barriers (B16-B19), provide empirical evidence and a clear mandate for the evolution of Egypt’s building regulatory framework. Viewed through a Multi-Level Governance (MLG) lens, these barriers highlight a critical disconnect between levels of authority. The current Egyptian Code for Energy Efficiency in Buildings (ECPS 306/2021) focuses effectively on operational energy at the national level but presents a significant gap by omitting embodied carbon, a major contributor to a building’s lifecycle emissions as highlighted by our respondents. Our results suggest that the code’s evolution should be envisioned as a multi-stage process to systematically address these identified gaps and improve coordination across governance levels.

An immediate enhancement would involve strengthening the code to address enforcement barriers (B17) at the local level by clarifying compliance pathways, verification protocols, and accountability mechanisms. Following this, the most direct application of our findings is the medium-term expansion of the code at the national level to integrate embodied carbon thresholds for key building materials and assemblies. This crucial update would directly address the lack of clear carbon reduction regulation (B16) and transform the market by creating mandatory demand for low-carbon materials, thereby overcoming the barrier of a market that prioritizes cost and speed over carbon reduction (B3). Ultimately, a long-term vision for the code should involve mandating a unified national methodology for whole-life carbon accounting. This would address the lack of a single regulating agency for carbon accounting (B19) by creating a clear lead institution and position the Egyptian regulatory framework to govern total carbon emissions (operational + embodied), aligning it with global best practices. Therefore, this study’s prioritization of legislative barriers does not merely identify a gap but provides a targeted, multi-level roadmap for policymakers at the Housing and Building National Research Center (HBRC) to update the ECPS 306/2021. Our barrier-solution map indicates that such a regulatory evolution (S7: Legislation), particularly when coupled with financial incentives (S2) that can be deployed across national and local authorities, is perceived by stakeholders as one of the most powerful levers for change in the Egyptian construction sector.

The policy recommendations arising from this study, focused on legislative evolution and financial mechanisms, are directly aligned with Egypt’s national commitments to sustainable development. The proposed expansion of the Egyptian Code to include embodied carbon is critical for achieving the decarbonization targets set out in Egypt’s Nationally Determined Contributions (NDCs). Furthermore, the focus on sustainable infrastructure, resource efficiency, and market transformation, driven by addressing top economic and legislative barriers (B3, B24, B16, B17), supports the economic transition goals outlined in Egypt’s Vision 2030, which seeks to promote sustainable infrastructure and resource efficiency. By tackling the top barriers, stakeholders can create a ripple effect, transforming industry norms and fostering an environment where economic and environmental priorities are balanced. This shift will not only support decarbonization goals but also promote long-term resilience and competitiveness within the construction sector. Lastly, this study represents a pioneering effort to explore and rank decarbonization barriers and solutions in construction projects in Egypt, while also capturing the perceptions of a diverse range of stakeholders involved in such projects. The findings of this research are particularly valuable for developing actionable recommendations to advance decarbonization and promote the use of sustainable building materials in construction projects within developing countries like Egypt. The contributions of this study aim to inspire decision-makers, researchers, and industry practitioners to design more comprehensive and practical solutions for overcoming decarbonization challenges. By providing a deeper understanding of the factors that influence decarbonization, this research offers a roadmap for targeted interventions that can enhance the adoption of sustainable construction practices.

## Study limitations

While the study offers valuable insights into the barriers to decarbonization in the construction industry, particularly in developing countries like Egypt, several limitations should be acknowledged to ensure a comprehensive understanding of the challenges and opportunities. Firstly, the sample size of 125 respondents, though substantial, may not fully represent the broader range of perspectives across the entire construction industry. The sample was obtained using selective sampling through LinkedIn, which likely introduced biases. Participants with LinkedIn profiles may represent a specific demographic, such as professionals in senior roles or those with active engagement in sustainability-related discussions. Consequently, the findings primarily reflect the perspectives of these sustainability-focused professionals, who are considered early adopters crucial for crafting regulatory strategies. We acknowledge that this selection means the findings do not represent all professionals in the Egyptian construction sector and may not reflect the experiences or views of those less engaged in sustainability or decarbonization efforts, which limits the immediate generalizability to the broader population. Future research could benefit from a more diverse sample that includes a wider range of roles within the construction industry, especially those less likely to be active on LinkedIn or involved in high-level decision-making. Another limitation is the cross-sectional nature of the study, which provides a snapshot of the barriers at a single point in time.

Additionally, the study focused on stakeholders and practices within the formal construction sector in Egypt. The study excludes Egypt’s informal construction sector, which represents a substantial share of national building activity in many developing contexts, and was not explicitly addressed. The barriers, solutions, and stakeholder dynamics identified may not fully apply to this segment, which often operates with different economic models, regulatory constraints (or lack thereof), and access to resources/information. Future research must address how decarbonization efforts impact and are impacted by the informal sector. While this study focuses on the systemic barriers to decarbonization, which often become most evident through challenges in reducing embodied carbon due to upfront costs and material selection, we acknowledge that optimal decarbonization strategies must adopt a whole-life carbon (WLC) perspective. Lützkendorf & Balouktsi^24^ emphasize that this approach involves carefully balancing embodied and operational carbon, a trade-off increasingly recognized in leading global sustainability frameworks such as LEED and BREEAM.

By diagnosing key barriers such as perceived costs, data gaps, and weak market demand, this study highlights the fundamental obstacles that impede not just embodied carbon reduction but also the sophisticated whole-life carbon analysis necessary for optimal decarbonization. To advance from barrier identification to solution implementation, future work must now develop dynamic WLC models that explicitly account for these Egyptian-specific challenges. This effort should be complemented by empirical case studies quantifying EC-OC trade-offs for local building types and by exploring financial mechanisms (e.g., green bonds, PPPs) to mitigate the economic barriers (B24, B28) that currently block WLC-positive investments. The framework established herein provides a critical roadmap for guiding these essential next steps.

A further limitation of this study is its focus on identifying and mapping barriers to solutions, rather than empirically testing the proposed solutions in real-world settings. While the research provides a critical, evidence-based roadmap for overcoming decarbonization challenges, a necessary foundational step, the practical efficacy, scalability, and unintended consequences of solutions like financial incentives (S2) or new legislation (S7) remain to be validated through pilot implementation and longitudinal case studies. This testing constitutes an essential and logical next step for future research, building directly upon the framework established here.

## Conclusions

The primary objective of this study was to identify and analyze the financial, technological, and regulatory challenges hindering decarbonization efforts in the construction sector. A comprehensive review of existing literature on barriers to reducing carbon emissions and adopting sustainable practices in the construction industry was conducted, revealing a set of key barriers. These barriers were systematically categorized into six groups for analysis. Subsequently, a review of potential solutions was undertaken, linking specific strategies to each of the 32 identified barriers.

The findings presented in this study underscore the pivotal role of government in driving the transition toward sustainable construction practices. For instance, B24 (perception of higher costs) within the Economic category emerged as the most critical barrier, emphasizing the need for government incentives and policies to offset these challenges. The second-ranked barrier, B3 (a limited market prioritizing cost and speed over reducing embodied carbon), in the Resources category, highlights the necessity of market transformation and demand stimulation for low-carbon solutions. Additionally, B9 (high initial investment costs for low-carbon technologies), also in the Economic category, further reinforces the need for financial support mechanisms to lower entry barriers for stakeholders. Barriers ranked fourth through sixth, namely B16, B17, and B18, fall within the Legislative category. These barriers point to the critical importance of establishing a supportive legislative framework to drive decarbonization efforts forward. Policies that mandate or incentivize sustainable practices can create the structural environment necessary to overcome these challenges and encourage industry-wide adoption. The study emphasizes that advancing decarbonization in the construction sector requires collective effort. It is not the responsibility of a single stakeholder but rather the combined contributions of all categories: economic, legislative, technical, and market-driven, to propel meaningful progress. By addressing these barriers holistically, the industry can move closer to achieving its sustainability and decarbonization goals.

The study also examined stakeholder perceptions of responsibility for minimizing embodied carbon emissions in construction projects, yielding insightful rankings. Government and regulatory bodies emerged as the top-ranked stakeholders, underscoring their critical role in establishing and enforcing a supportive legislative framework to drive decarbonization. This result reflects the industry’s reliance on clear regulations, policies, and mandates to prioritize sustainability goals effectively. Sustainability consultants and architects were ranked second, emphasizing their pivotal role in translating legislative goals into actionable strategies through technical expertise, innovative design approaches, and sustainable material selection. This highlights the collaborative nature of decarbonization efforts, where technical teams act as vital intermediaries in operationalizing policy directives on construction sites. In terms of solutions for decarbonization, the ranking revealed that financial incentives were perceived as the most effective measure, addressing five key barriers. Furthermore, our analysis revealed that power imbalances, conflicting priorities between development and sustainability goals, and institutional resistance are critical underlying socio-political barriers that must be managed through inclusive policy design and targeted communication strategies.

Incentives such as subsidies, tax credits, and grants are seen as powerful tools to reduce the economic burden of adopting low-carbon technologies and practices. Legislation followed closely, also addressing five barriers, further affirming its foundational role in driving systemic change. However, addressing the scale and complexity of economic barriers, particularly large upfront capital requirements and project risks, often requires mobilizing private finance beyond traditional public funding. Financial models such as Public-Private Partnerships (PPPs) offer a complementary approach. Casady, Cepparulo and Giuriato^25^ argue that public-private partnerships (PPPs) can leverage private sector capital, expertise, and efficiency for infrastructure development and service delivery, sharing risks and responsibilities between public and private entities. While, Qadir and Pillay^26^ highlight that other innovative mechanisms like green bonds or blended finance structures can pool capital from diverse investors specifically for low-carbon projects, potentially lowering financing costs and mitigating risks that deter private investment.

A primary policy recommendation arising from this study is for regulatory bodies (HBRC and EEAA) to initiate a process to expand the scope of the Egyptian Code for Improving the Efficiency of Energy Use in Buildings (ECP 306–2017) to include embodied carbon. Furthermore, the EIA process under Law No. 4 of 1994 should be updated to incorporate whole-life carbon accounting for major developments. These actions would directly mitigate the top-ranked legislative and economic barriers by creating a regulated and transparent market for low-carbon solutions. These findings underline the dual importance of economic and regulatory interventions as complementary strategies for overcoming decarbonization barriers. Notably, legislation consistently emerged as the highest-ranking factor across all survey questions, reflecting its perceived significance in shaping industry behavior and providing the necessary structure for sustainable transformation. This result highlights a critical call to action for policymakers to craft well-rounded and enforceable legislative frameworks that incentivize decarbonization while penalizing unsustainable practices. The combined emphasis on government leadership, financial mechanisms, and technical expertise indicates that effective decarbonization is a multi-stakeholder endeavor. Government bodies must lead by setting ambitious yet achievable targets, while architects and consultants must champion innovation and sustainability in project execution. The alignment of legislative support with economic incentives could serve as a catalyst for widespread adoption of low-carbon construction practices, creating a ripple effect across the value chain.

## Data Availability

The datasets supporting this study are available from the corresponding author upon reasonable request.
